# Safety and Tolerability of *Lactobacillus reuteri* DSM 17938 and Effects on Biomarkers in Healthy Adults: Results from a Randomized Masked Trial

**DOI:** 10.1371/journal.pone.0043910

**Published:** 2012-09-06

**Authors:** Nisha Mangalat, Yuying Liu, Nicole Y. Fatheree, Michael J. Ferris, Melissa R. Van Arsdall, Zhongxue Chen, Mohammad H. Rahbar, Wallace A. Gleason, Johana Norori, Dat Q. Tran, J. Marc Rhoads

**Affiliations:** 1 Department of Pediatrics, University of Texas Medical School at Houston, Houston, Texas, United States of America; 2 Department of Pediatrics, Louisiana State University Health Science Center and The Research Institute for Children, New Orleans, Louisiana, United States of America; 3 Biostatistics/Epidemiology/Research Design Core, Center for Clinical and Translational Sciences, University of Texas Health Science Center at Houston, Houston, Texas, United States of America; 4 Division of Epidemiology, Human Genetics, and Environmental Sciences, The University of Texas School of Public Health at Houston, Houston, Texas, United States of America; Université d'Auvergne Clermont 1, France

## Abstract

**Background:**

There are few carefully-designed studies investigating the safety of individual probiotics approved under Investigational New Drug policies.

**Objectives:**

The primary aim of this prospective, double-blind placebo-controlled trial was to investigate if daily treatment of adults with *Lactobacillus reuteri* DSM 17938 (LR) for 2 months is safe and well-tolerated. Our secondary aim was to determine if LR treatment has immune effects as determined by regulatory T cell percentages, expression of toll-like receptors (TLR)-2 and −4 on circulating peripheral blood mononuclear cells (PMBCs), cytokine expression by stimulated PBMC, and intestinal inflammation as measured by fecal calprotectin.

**Methods:**

Forty healthy adults were randomized to a daily dose of 5×10^8^ CFUs of LR (n = 30) or placebo (n = 10) for 2 months. Participants completed a daily diary card and had 7 clinic visits during treatment and observation.

**Results:**

There were no severe adverse events (SAEs) and no significant differences in adverse events (AEs). There were no differences in PBMC subclasses, TLRs, or cytokine expression after treatment. The probiotic-treated group had a significantly higher fecal calprotectin level than the placebo group after 2 months of treatment: 50 µg/g (IQR 24–127 µg/g) vs. 17 µg/g (IQR 11–26 µg/g), p = 0.03, although values remained in the normal clinical range (0–162.9 µg/g). LR vials retained >10^8^ CFUs viable organisms/ml.

**Conclusions:**

LR is safe and well tolerated in adults, without significant changes in immunologic markers. There was a small but significant increase in fecal calprotectin, perhaps indicating some element of immune recognition at the intestinal level.

**Trial Registration:**

Clinical Trials.gov NCT00922727

## Introduction

In recent years, there has been increasing clinical evidence indicating beneficial effects of probiotics in the prevention and/or treatment of gastrointestinal diseases. Probiotics are live microorganisms, that when ingested, confer a health benefit on the host [1]. Probiotics are generally given as supplements of commensal microbiota. There is emerging clinical evidence that *Lactobacillus reuteri* (LR) may be promising as a treatment for infantile colic [2] and diarrheal disease [3]. LR is a commensal organism that has been isolated from breast milk, and is able to colonize the human gastrointestinal tract, specifically the gastric body and antrum, duodenum and ileum [4].

Lactobacillus species are generally not considered pathogenic; however, probiotics have been implicated in rare cases of endocarditis and bacteremia in immunocompromised patients [5]. There are reports of infections directly linked to the ingestion of probiotic products [6]–[9], and one multicenter trial of a probiotic for the treatment of patients with severe pancreatitis documented an increase in mortality in the treated group [10]. Recently, a large project focused on the safety of probiotics was conducted by the Southern California Evidence-based Practice Center (EPC) under contract to the Agency for Healthcare Research and Quality (AHRQ). The AHRQ report literature search identified 11,981 publications, of which 622 studies were included in the review. Of these, 387 studies reported specific adverse events. Across all included studies and treatment arms, 24,615 participants used a probiotic. The authors concluded that in the randomized control trials there was “no evidence that the quantity of reported adverse events was increased in short-term probiotic intervention arms compared to control groups.” However, the report went on to state that “There is a lack of assessment and systematic reporting of adverse events in probiotic intervention studies… and the current literature is not well equipped to answer questions on the safety of probiotic interventions with confidence” [11].

The primary aim of this Phase I prospective, double-blind placebo-controlled trial was to provide evidence that treatment of adults with *Lactobacillus reuteri* DSM 17938 for 2 months is safe and well-tolerated. Our long-term goal in staging the Phase I/II RCT design from adults to risk groups of children and infants is to demonstrate that a two-month course of *Lactobacillus reuteri* DSM 17938 is a safe and effective therapy for infantile colic. Development of this probiotic intervention under the US Food and Drug Administration, Center for Biological Evaluation and Research [FDA/CBER] oversight regulates for ‘fit for intended purpose’ product labeling and improved quality control. *L. reuteri* is currently marketed as a nutritional supplement. However, purported health claims have not been substantiated in the United States for adults or children.

Our secondary aim focused on describing potential immunologic responses to ingestion of the probiotic *Lactobacillus reuteri*. Specifically, we explored the effects of LR treatment on regulatory T cells (Tregs), toll-like receptors (TLR)-2 and-4 expressions on circulating peripheral blood mononuclear cells (PBMCs), and cytokine expression by stimulated PBMCs. The mechanism of LR's action conferring benefit to the human host remains under investigation. LR produces a compound, reuterin, with antimicrobial properties capable of inhibiting a wide spectrum of other microorganisms, including pathogens. We and others have shown that LR reduces inflammation in intestinal cells and tissues *in vitro* [12], [13], that LR reduces inflammation in the intestine of lipopolysaccharide-fed newborn rat pups [14], and LR reduces the incidence and severity of experimental necrotizing enterocolitis via modulation of TLR4 and NF-κB signaling in the intestine [15].

The gastrointestinal mucosa is exposed to billions of non-pathogenic, commensal organisms. The mechanisms for “tolerability” of the host to these organisms and the interplay between the commensals and the host immune response are of obvious interest. Tregs, particularly the naturally occurring CD4^+^Foxp3^+^ cells, have emerged in recent years to have key roles in maintaining self-tolerance. TLRs are proteins that are part of a family of pathogen recognition receptors expressed on both immune cells such as B-cells, dendritic cells, and monocytes (PBMCs), and non-immune cells (epithelial cells and endothelial cells). There are 10 TLRs currently recognized in humans; each recognizes a particular pathogen-associated molecular pattern, or PAMP. TLR2 recognizes lipoproteins such as peptidoglycan found in bacterial cells walls. TLR4 is the main receptor for lipopolysaccharide. Different probiotic bacteria are known to interact with distinct TLRs [16]. In vitro studies have suggested that the interaction of the probiotic strain *Lactobacillus casei* with intestinal epithelial cells is mediated by TLR2 [17]. However, a mouse model of colitis has demonstrated that lactic acid bacteria may improve colitis via inhibition of TLR4- mediated NF-κB activation [18]. In another mouse model of colitis, oral ingestion of *Lactobacillus casei* alleviated colitis and increased the suppressive function of CD4^+^Foxp3^+^ Tregs [19]. Secondary aims also included determination of the effect of LR on fecal calprotectin, a marker for intestinal inflammation as well as to describe major shifts in the overall fecal microbial community [20]

## Subjects and Methods

### Ethics statement

The procedures followed were in accordance with the ethical standards of The University of Texas Health Science Center at Houston after approval by the Institutional Review Board of University of Texas Health Science Center and Memorial Hermann Hospital (IRB # HSC-MS-08-266). Written informed consent was obtained from all participants prior to enrollment in the study. The protocol for this trial and supporting CONSORT checklist are available as supporting information; see Protocol S1 and Checklist S1.

### Study participants

**Figure 1:**
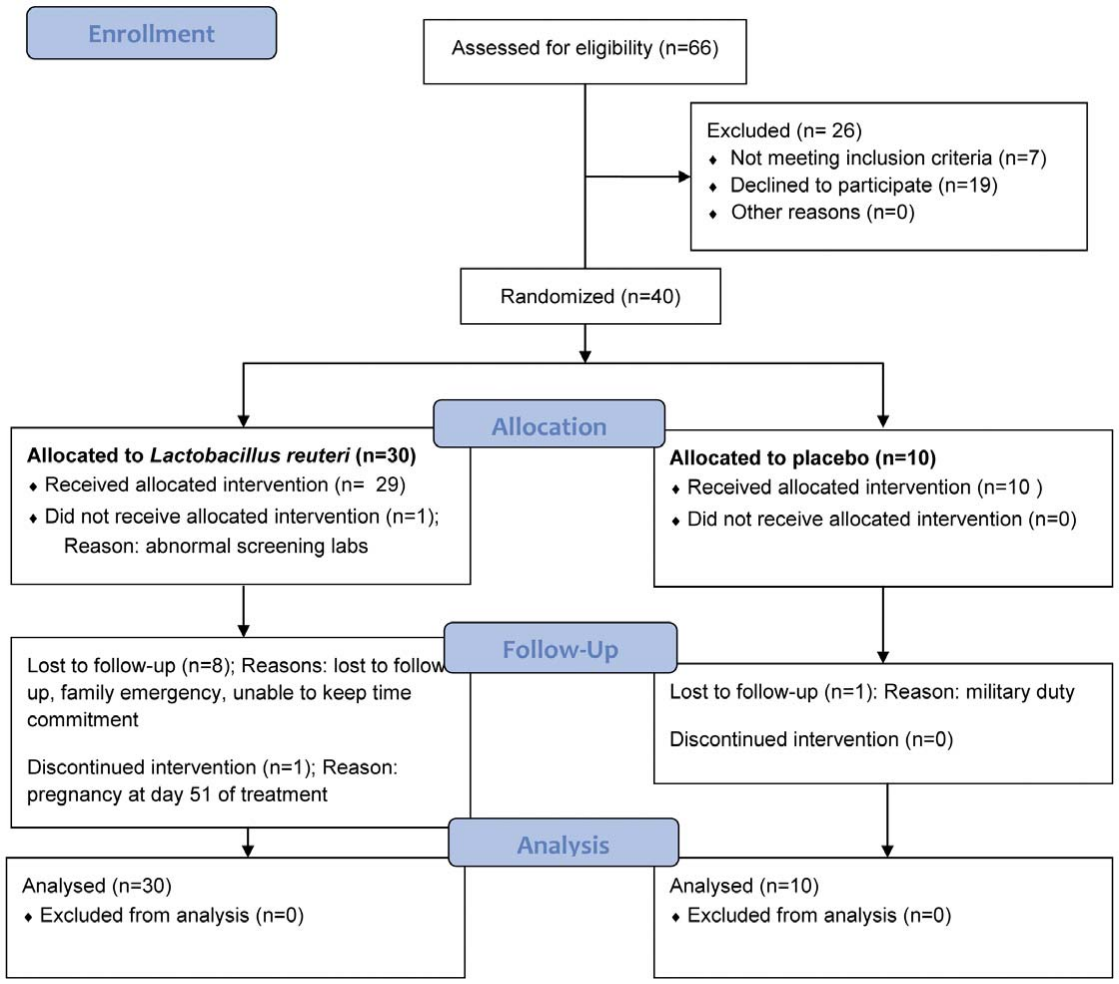
Participant flow diagram. ITT = Intent to Treat.

Eighty-eight patients were screened for eligibility. See **Table 1** for complete listing of inclusion and exclusion criteria. Forty healthy volunteers were enrolled in the study. See **Figure 1** for participant flow diagram. Only two study participants were allowed per household.

**Table 1:** Inclusion and exclusion criteria.

INCLUSION CRITERIA	EXCLUSION CRITERIA
Healthy male and non-pregnant female adults	Pregnancy or breastfeeding
Ages 19–60	Immunosuppressive medications, including oral corticosteroids
No other recognized illness	Positive result of HIV, hepatitis B, and/or hepatitis C antibody screening
	Blood parameters outside the normal range deemed to be clinically significant
	Gastrointestinal-related diseases and surgeries
	Antibiotic allergy
	Use of probiotics during the 90 days prior to screening
	Diarrheal illness within 30 days prior to screening
	Use of oral antibiotics or anti-fungals within the 2 weeks preceding screening
	Current use of oral laxatives; alcohol use of more than 2 drinks per day
	Implanted prosthetic devices (e.g. prosthetic heart valves)
	Known sensitivity to sunflower oil or products containing linolenic/oleic acids
	Unwillingness to forego ingestion of any other probiotic-containing products, including yogurt supplemented with probiotics during the 6-month study period
	Presence of fever or a pre-existing adverse event monitored during the study

### Study design

This study was a single center, randomized, double-blind, placebo-controlled safety trial designed to evaluate the safety of treatment with *Lactobacillus reuteri* in healthy adults, conducted between October 2009 and July 2011. Oversight approval and monitoring was also provided by a Data Safety Monitoring Board (DSMB), the Food and Drug Administration/Center for Biologics Evaluation and Research (FDA/CBER) [IND #13561], and the National Institutes of Health/National Center of Complementary and Alternative Medicine (NIH/NCCAM) Program Officials and Office of Clinical and Regulatory Affairs (OCRA) prior to enrollment of subjects.

At time of “screening visit” (day 0), participants signed informed consent, underwent physical examination by one of three physician-investigators and submitted venous blood samples to ensure general health and eligibility for the study. Venous blood sample measurements included complete metabolic panel (CMP), complete blood count (CBC), C-reactive protein (CRP), HIV serum antibody, hepatitis B surface antigen, and hepatitis C antibody. Participants agreed to forego any other probiotic-containing product during the study period. Subjects were informed about their eligibility by the study coordinator within 2 days of the screening visit. A “baseline visit” was subsequently scheduled with physician-investigator and study coordinator to assure general health of the subject, to deliver study product, and to obtain research protocol labs (i.e. samples for cytokine and flow cytometric analysis). At time of the baseline visit (day 1 of treatment), subjects eligible for participation (n = 40) were randomly allocated to receive either the probiotic, *Lactobacilllus reuteri*, or the placebo product (sunflower oil) with a ratio of 3∶1 (i.e., 30 were assigned to the treatment arm and 10 were assigned to the placebo arm). The participants were instructed to take the product daily for a period of 2 months.

As assessment of safety and tolerability was the primary objective of this trial, participants underwent frequent evaluations by physician-investigators throughout the treatment period of two months and for four months following treatment (observation period). Safety was assessed by physical examination and laboratory markers obtained at clinic visits occurring at two week intervals during treatment phase of the study. All clinic visits took place at the Clinical Research Unit (CRU) of The University of Texas Health Science Center at Houston. Strict monitoring of adverse events reported by patients was recorded throughout the study period in compliance with the FDA Adverse Events Response System [AERs], as well as monitoring using a severity clinical index. Participants were also instructed to complete a daily diary of any adverse events during the two months.

**Figure 2:**
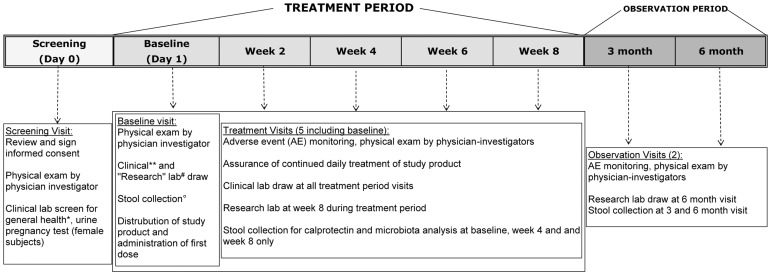
Study design. *Clinical labs at screening included complete blood count (CBC), comprehensive metabolic profile (CMP); HIV and hepatitis B and C serology. **Clinical labs at baseline and subsequent visits included CBC, basic metabolic profile (BMP), and urine. ^#^ “Research” labs included whole blood for Treg, cytokine, and TLR analyses. ° Stool collection obtained for microbiota and calprotectin analysis.

Participants continued to report all health-related events and were again evaluated by physician-investigators in the observation period. This occurred 1 month after treatment (3 month visit) and 4 months after treatment (6 month visit). Blood samples for health and safety screens were collected at all treatment and observation visits. Fecal samples were collected at baseline visit, after 1 month of therapy and at end of the two-month treatment period. Fecal samples were also collected at both observation period visits. See **Figure 2** for a schematic representation of clinic visits, serum and fecal laboratory collection times. Quality assurance of research procedures were regularly assessed by our institutional IRB, DSMB and the NIH. *Study Product (Intervention).* Permission to pursue investigational studies of this probiotic was granted by the United States Department of Health and Human Services, FDA (IND number BB 13561). Subjects were instructed to take 5 drops of study product (probiotic or placebo) daily. Each drop of study product contained 1×10^8^ colony-forming units (CFUs) *L. reuteri* DSM 17938 per drop, yielding a total daily dose of 5×10^8^ CFUs. The oil drops were manufactured by BioGaia, Inc., Stockholm, Sweden. The placebo product (provided by BioGaia) contained sunflower oil alone. Study products (probiotic and placebo) were placed in vials with the same appearance. Until May 2010, each study vial contained 5 ml of study product, approximately equal to a 2-week supply. After May 2010, the company provided vials that contained 10 ml of treatment or placebo product. Participants were instructed to bring in their vials to each follow-up visit, and remaining product was measured to assure compliance with therapy. One laboratory investigator was unblinded to the study groups in order to confirm viability of the probiotic in 1 of every 5 treatment group patients. The pharmacy staff was responsible for storing and dispensing the product and placebo according to the randomization schedule provided by the study statistician. All investigators directly involved in this randomized control trial were blinded to identifying subject allocation in the safety/adverse events reporting data.

### Sample size

Because this was a safety and tolerability study, we determined the sample size in such a way that if the risk estimates for adverse events (AEs) associated with LR exceeded twice that of the general population with 95% confidence, then the study would be halted for the DSMB review. A panel of experts was assembled to provide reasonable risk estimates for fever, diarrhea, vomiting, mild illness with positive blood culture, and sepsis as potential AEs associated with LR. Assuming that the daily incidence rates of AEs (fever, diarrhea, vomiting, mild illness with positive blood culture, and sepsis) in the general population are about (1/160, 1/200, 1/365, 1/1000, and 2/10000), respectively, and all the participants were followed for 56 days, we determined that 30 participants in the LR treatment arm would provide 1680 days of observation that is sufficient for the stopping rules to detect the aforementioned acceptable risks with 95% confidence. For example, for 30 participants (1680 days of observation) who received the *LR* we determined the cut-off incidence of 29, 24, 15, 7, and 3 for fever, diarrhea, vomiting, mild fever with positive blood culture, and sepsis, respectively. Because we reported every 10 participants to the DSMB, we developed similar cut-off incidence values for every 10 participants and cumulatively. The decision to enroll 10 participants in the placebo arm was to collect some preliminary data for future studies. We acknowledge that 30 participants in the *LR* arm and 10 participants in the placebo arm may not provide adequate power (e.g., 80%) to detect reasonable effect sizes for many secondary variables collected in this study.

### Subject randomization/blinding

A block randomization schedule was prepared by the study biostatistician, enabling the research pharmacist to assign each subject with a randomization number allocated to the treatment vs. placebo group. Participants were randomized to the treatment and placebo groups in a 3∶1 ratio (block allocation), with a total of 40 participants (30 in the treatment arm and 10 in the placebo arm) aimed to detect differences in safety. This study was fully blinded with respect to all clinical investigators, research nurses, and subjects until the completion of the study. The unblinded investigator was blinded to the safety/adverse events data.

### Statistical analysis

For baseline comparisons, we provided appropriate descriptive statistics. Specifically, for continuous variables we examined the distributions and reported mean and standard deviations if the distributions were approximately normal. For variables with skewed distributions we identified suitable transformations to normalize the distribution. If we did not succeed in finding a suitable transformation or reported medians along with Interquartile Ranges (IQR). For the comparison of the primary safety outcomes we followed principles of an intent-to-treat analysis. Since this trial was not powered for efficacy, the analysis of the secondary outcomes were considered exploratory, hence no adjustments were made with respect to the multiple comparisons and multiple testing. However, whenever appropriate, we used t-test and Fisher exact or Mann-Whitney U-test to continuous and categorical variables, respectively. We also calculated 95% CIs for the mean difference between the two study arms. In addition, we calculated effects sizes for the secondary outcomes by dividing the mean differences by the pooled standard deviation between the two study arms. All the analyses were conducted by using statistical software SAS 9.2 (SAS Institute, Cary, NC).

### Outcome measures

The primary outcome measures of this study were to assess the safety and tolerability *of Lactobacillus reuteri* in healthy adults. Safety was assessed by strict monitoring of serious adverse events (SAE) and adverse events (AE) reported by patients and through scheduled visits conducted by clinicians. Serious adverse events (SAE) were defined as any event which resulted in death, a life-threatening adverse experience, inpatient hospitalization or prolongation of existing hospitalization, a persistent or significant disability/incapacity, or a congenital anomaly/birth defect. AEs were defined as any untoward medical occurrence in a subject receiving LR. An AE could include a clinically significant abnormal laboratory finding, symptom, or disease temporally associated with the use of a medicinal product, whether or not considered related to the medicinal product. Patients were instructed to complete a diary card each day with description of all symptoms experienced during the treatment phase. As symptoms occurred, they were reported by phone to the study coordinator and/or primary investigator and were recorded in the respective patient study charts. Symptoms monitored as adverse events on the diary cards included nausea, vomiting, diarrhea, headache, rash, and acute systemic allergic reactions. Fecal samples were collected for measurement of LR in the stool specimens by quantitative real-time PCR (qPCR) analysis of the 16S rRNA gene of LR, assessment of bacterial composition of stool specimens by PCR amplification of 16S rRNA genes, and analysis of banding pattern on denaturing gradient gel electrophoresis (DGGE). Fecal samples for these analyses were obtained at the following time points: baseline visit, after 2 weeks of treatment, end of treatment period, and at the two observation period visits – 3 months and 6 months.

Secondary outcomes provided data regarding the effect of *Lactobacillus reuteri* on the immune system. These “research labs” or blood samples obtained for flow cytometric analysis for quantification of PBMCs, and for TLR and cytokine expression by stimulated PBMCs were taken at the baseline clinic visit, 2 month clinic visit (end of treatment) and the 6 month observation period visit. Measurement of subacute intestinal inflammation was obtained via fecal calprotectin at the baseline visit (prior to ingestion of the product), one month of treatment, and at end of the 2-month treatment period. Fecal calprotectin was also assessed in the observation period at the 3 month visit and the 6 month visit.

### Research lab protocols

Materials and Methods S1 provides detailed materials and methods for measuring the expression of TLRs, percentage of Tregs in PBMC by flow cytometric analysis, cytokine production by stimulated PBMC, fecal calprotectin, and microbiota analysis.

### Assessment of safety and tolerability

All symptoms reported by participants were recorded. Each subject completed a symptom diary card for solicited symptoms that was turned in at every clinic visit. Adverse events (AE) were coded using the Medical Dictionary for Regulatory Affairs (MedDRA) coding dictionary. Treatment-emergent AEs were defined as any event with a start date occurring on or after first day of treatment; or, if pre-existing, worsening after first day of treatment. If a subject reported the same AE more than once, then that subject was counted only once for the summary of that AE, using the most severe intensity. The same protocol for reporting applied for significant adverse events.

A Data and Safety Monitoring Board (DSMB) consisting of specialists in Infectious Disease (Pediatric and Internal Medicine), Gastroenterology, and Statistics also monitored the safety of the subjects in this trial. The DSMB members were not otherwise involved in this RCT. The DSMB met after every 12 patients completed two months of therapy if no significant complications arose.

### Protocol deviations

There were four protocol deviations in the entire study period. All of these deviations were reported to the DSMB. The deviations included the following: one inadvertent “research” blood draw when it was not indicated, one patient stopped treatment at day 51 as she became pregnant, one patient had a study visit outside of scheduled appointment window, and one patient ran out of study drug for two days before additional drug product was provided. None of these protocol deviations resulted in expulsion from the study. All were included in the intention to treat analysis.

## Results

### Subjects

Forty subjects aged 19–60 were enrolled in this safety study. The recruitment period was from October 29, 2010–March 21, 2011. The follow-up period was from January 25, 2010–July 18, 2011. The trial ended after all enrolled subjects completed the six month study period. There were a total of thirty patients were in the treatment (LR) group and 10 patients in the placebo treated group. All patients were included for comparison of the primary safety outcome following the principles of intent-to-treat. Thirty-one patients completed the entire 6 month study period (Figure 1). Nine patients left the study prematurely; two relocated, four left for personal reasons, and three were lost to follow-up (Figure 1). Of these, only one of the withdrawals was from the placebo group (relocation). Only two participants were from the same household. There were no differences between the treatment and placebo groups with respect to baseline characteristics including gender, age, ethnicity, weight, height, BMI, vital signs, white blood cell count (WBC), glucose, blood urea nitrogen (BUN), or CRP (**Table 2**). Similarly, there were no differences comparing the two study groups with respect to baseline “research” laboratory characteristics, including percentages of PBMC subtypes, TLR expression on PBMC, cytokine expression by stimulated PBMC, and fecal calprotectin (t-test and Kruskal-Wallis test performed for values with normal and abnormal distributions respectively. None of the p-values were <0.05) (**Table 3**).

**Table 2:** Baseline clinical characteristics of the study groups.

	Probiotic group	(n = 30)	Placebo group	(n = 10)	
	Mean	SD	Mean	SD	p-value
Age (yrs)	34.6	12.1	32.9	10.2	0.8*
Female (n, %)	13	43%	4	0.4	1.0
Ethnicity (n, %)					
Asian	2	7%	2	20%	
Black	8	27%	2	20%	
Hispanic	4	13%	0	0%	
White, not-Hispanic	16	50%	6	60%	
Weight (kg)	81.8	16.7	79.6	12.4	0.7
Height (cm)	170.5	14.8	169.3	11.3	0.8
BMI (kg/m^2^)	28.3	5.9	27.7	2.6	0.5*
Pulse rate (beats/min)	73.4	12.1	70.0	9.3	0.4
Blood pressure (mmHg)					
Systolic	126	13	125	8	0.85
Diastolic	74	11	72	7	0.98*
Temperature (C°)	36.8	0.2	36.8	0.3	0.9
WBC (×10^3^/µl)	5.9	1.4	6.3	1.4	0.5
Lymphocytes (%)	29	9	32	9	0.45
Glucose (mg/dL)	88.1	14.4	79.9	13.3	0.1*
hsCRP (mg/L)	1.06	1.6	1.20	1.89	0.71*
BUN (mg/dL)	13.0	4.2	14.8	4.4	0.3

Abbreviations: Body Mass Index (BMI), White blood cell count (WBC), highly sensitive C-reactive protein (hSCRP), Blood urea nitrogen (BUN).

* denotes p values obtained from non-parametric Kruskal-Wallis test.

**Table 3:** Baseline research laboratory characteristics of the study groups*.

	Probiotic group (n = 30)		Placebo group (n = 10)		
Peripheral Blood Mononuclear Cells (PBMCs)					
	Mean	SD	Mean	SD	p-value
Monocytes (%)	7.1	2.3	7.3	2.2	0.87
mDC (%)	0.17	0.09	0.24	0.15	0.17
pDC (%)	0.14	0.09	0.17	0.09	0.41

	Median	IQR	Median	IQR	
Tregs (%)	6.0	4.9–7.0	5.9	3.6–8.5	0.72
Lymphocytes (%)	23	19–23	22	20–30	1.00
B cells (%)	9.5	7.8–12.2	10.2	6.0–12.8	0.75

Cytokine Production by Stimulated PBMC (pg/ml) **					
	Median	IQR	Median	IQR	p-value
GMCSF	22	10–33	15	4–28	0.54
IFNγ	3440	1258–11638	9452	242–2736	0.61
IL10	1.4	0.6–2.2	1.6	0.5–3.2	0.85
IL12	1.2	0.8–2.3	1.2	0.7–2.2	0.74
IL1β	7.8	4.8–18.4	10.7	3.7–84.5	0.80
IL2	208	34–757	292	56–5571	0.63
IL6	3.8	1.8–10.3	3.2	1.1–7.4	0.70
IL8	3697	951–10782	1450	144–5623	0.25
TNFα	131	46–352	140	16–705	0.92

TLR Expression in PBMC (MFI)					
	Median	IQR	Median	IQR	p-value
Monocyte TLR2	11	9–17	17	12–21	0.19
Monocyte TLR4	8.0	5.8–16.4	15.3	9.2–26.3	0.11
B cell TLR2	5.1	2.7–7.5	8.1	4.2–11.0	0.18
B cell TLR4	3.1	2.5–5.3	5.1	2.7–11.1	0.29
mDC TLR2	6.0	4.9–8.9	7.4	2.7–14.4	0.77
mDC TLR4	4.2	3.3–6.8	5.7	5.7–8.9	0.90
pDC TLR2	10	5–15	12	3–23	0.63
pDC TLR4	5.4	3.9–7.8	10.3	1.2–12.4	0.47
Fecal Calprotectin (µg/g)	30	11–57	24	14–36	0.88

Abbreviations: T regulatory cells (Tregs), myeloid dendritic cells (mDC); plasmacytoid dendritic Cells (pDC); interferon-gamma (IFNγ); interleukin (IL); tumor necrosis factor-alpha (TNFα); toll-like receptor (TLR); mean fluorescence intensity (MFI).

* Mean, standard deviations with p-values based on t-tests reported for values following a normal distribution. Median, interquartile ranges (IQR) with p-values based on Kruskal-Wallis test reported for values with skewed distribution.

** PBMC (1×10^6^) stimulated by PMA (50 ng/ml) and inomycin 1 (µg/ml) for 16 hours.

### Product stability

We monitored product viability by measuring the remaining volume in the first and final containers of every 5^th^ patient throughout the treatment period of the study. We documented an average of 4.6×10^8^ CFUs of LR in the 9 screened patients receiving study product. All samples contained >1×10^8^ CFUs per day at the end of the active phase of the 2-month trial. Product compliance based upon returned vial volume in the group of subjects in the per-protocol analysis was 88%.

### Safety

Participants underwent physical examination and screening laboratory testing at baseline. All adverse events reported to the study coordinator and physician-investigators were recorded in the chart. A Data and Safety Monitoring Board (DSMB) evaluated all reported adverse events every 12 patients after completion of 2 months of treatment. All reported adverse events are shown in **Table 4**. There were no serious adverse events reported during the entire study period. Common everyday symptoms were reported in both groups, but there was no increased risk of adverse events or differences in the adverse events reported in the probiotic versus the placebo group (p = 0.46). None of the reported adverse events were determined to be related to the probiotic intervention by the independent DSMB members.

**Table 4:** Distribution (frequency, percentage) of the adverse events by study arm and type during treatment phase.

	Probiotic group (n = 30)			Placebo group (n = 10)		
Symptom	Mild, n (%)	Moderate, n (%)	Severe, n (%)	Mild, n (%)	Moderate, n (%)	Severe, n (%)
Nasal congestion	4 (13)	3 (10)	0 (0)	1 (1)	1 (1)	0 (0)
Headache	1 (3)	3 (10)	0 (0)	0 (0)	0 (0)	0 (0)
Sore throat	0 (0)	0 (0)	0 (0)	1 (1)	0 (0)	0 (0)
Nausea	2 (7)	0 (0)	0 (0)	0 (0)	0 (0)	0 (0)
Weight loss	1 (3)	0 (0)	0 (0)	0 (0)	0 (0)	0 (0)
Vomiting	7 (2)	0 (0)	0 (0)	0 (0)	0 (0)	0 (0)
Decreased appetite	3 (1)	0 (0)	0 (0)	1 (1)	0 (0)	0 (0)
Tongue numbness	0 (0)	0 (0)	0 (0)	1 (1)	0 (0)	0 (0)
Loose stools	2 (7)	0 (0)	0 (0)	0 (0)	0 (0)	0 (0)
Vaginal odor	1 (3)	0 (0)	0 (0)	0 (0)	0 (0)	0 (0)
Flatulence	1 (3)	0 (0)	0 (0)	0 (0)	0 (0)	0 (0)
Dry skin	1 (3)	0 (0)	0 (0)	0 (0)	0 (0)	0 (0)
Rhinnorhea	0 (0)	0 (0)	0 (0)	1 (1)	0 (0)	0 (0)
Watery eyes	0 (0)	0 (0)	0 (0)	1 (1)	0 (0)	0 (0)

One patient withdrew from study after randomization before receiving treatment.

None of the adverse events were identified to be related to treatment group by an independent data safety monitoring board (DSMB).

No significant difference in adverse events between groups (p = 0.46)

### Changes in the overall bacterial composition in stool specimens

**Figure 3:**
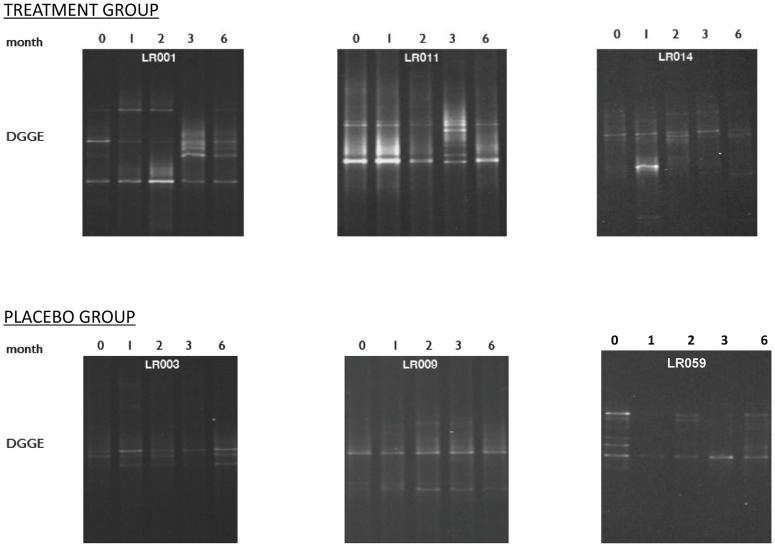
Representative DGGE images. Representative denaturing gradient gel electrophoresis images from six patients, three treated with LR (Treatment Group LR001, LR011, LR014) and three with placebo (Placebo group LR003, LR009, LR059). Each lane in a gradient gel shows bands of 16S rDNA detected in each of five stool specimens collected at 0, 1, 2, 3 and 6 months. Each band represents a different bacterial 16S rDNA sequence, i.e. a different bacterial taxon. Time 0 stools were collected before patients began consuming LR or placebo. Time 1 and 2 stools were collected after patients had been consuming LR or placebo for one and two months respectively. Time 3 and 6 stools were collected one month and four months after patients had discontinued consuming LR or placebo. For each patient, differences in band patterns across sampling time points indicate changes in the composition of bacteria, over time, in their stools. For example, Patient LR001 exhibited a notable change in stool bacterial composition between Time 0, a stool collected before consuming any LR, and Time 1, a stool collected after consuming LR for one month. Likewise, the placebo treated Patient LR059, exhibited a notable change in stool bacterial composition over the same interval. In contrast LR treated Patient LR011 exhibited no change in band pattern over that interval. We compared the total number of band pattern changes over each interval in the LR treated and placebo treated groups and found no significant difference in the influence of LR treatment compared to placebo treatment on changes in stool bacterial composition over any interval examined.

DGGE analyses were performed to evaluate changes in the overall composition of fecal microbiota over time and are presented in **Figure 3**. From baseline to 2 months, there was an observable change in microbiota pattern in 17 of 22 LR-treated subjects, but there was also a change in DGGE pattern in 6 of 10 placebo-treated subjects over the same interval. Similar findings were noted at 2, 3, and 6 month intervals. We found no statistical difference in the effect LR on bacterial composition in stool specimens compared to the placebo-treated controls over any time interval.

### Change in LR concentration in stool specimens

The results of the qPCR analysis of LR in stool specimens are summarized in **Table 5**. There was a marginally significant difference in the concentration of LR in stool specimens compared to placebo after 1 month of treatment (p = 0.06) and after 2 months of total therapy (p = 0.08). However, there was no difference in the concentration of LR in stool specimens compared to placebo treated control patients at the observation period visits. In general, our results showed very low numbers of LR at all visits.

**Table 5:** Median concentration of LR in stool specimens at various time points*.

Visit	Placebo	LR	*p*-Value
	Median (Q1, Q3)	Median (Q1, Q3)	
Baseline	0 (0, 0)	0 (0, 1.61)	0.19
1 Month	0.19 (0, 11.1)	11.85 (0.54, 118)	0.06
2 Month	0 (0, 2.17)	10.66 (0.37, 79.2)	0.09
3 Month	0 (0, 27.3)	0 (0, 0.58)	0.36
6 Month	0 (0, 0.40)	0 (0, 1.71)	0.94

* Copies of LR 16 s rRNA gene detected in 5 ng of stool.

Q1 represents the 25^th^ percentile.

Q3 represents the 75^th^ percentile.

### PBMC studies and TLR expression by PBMCs

Blood samples obtained for flow cytometric analysis for PBMCs and quantification of TLR expression by PBMC were taken at baseline, 2 months of treatment, and 6 month observation period visit. We found no differences in percentage of circulating lymphocytes, monocytes or dendritic cells, comparing the study groups. We were particularly interested in the regulatory T cell (Treg) subset of lymphocytes. We detected no significant differences in this cell population between the groups before or after treatment (**Table 6**). For TLR expression analyses, mean fluorescence intensity (MFI) was evaluated, corrected for isotype controls. There was no significant difference in TLR expression on any PMBC evaluated, comparing the probiotic treated and placebo groups (Table 6).

**Table 6:** Comparisons between study arms after 2 months of treatment.

	Probiotic group			Placebo group				
Peripheral Blood Mononuclear Cells *	n	Mean	SD	n	Mean	SD	95% CI**	Estimated Effect Size
Tregs (%)	23	6.32	2.40	10	6.91	2.34	−2.46,1.28	−0.25
Lymphocytes (%)	23	23.06	8.36	10	23.89	6.52	−6.67,5.00	−0.13
Log-transformed variables:								
B cells (%)	23	2.30	0.44	10	2.31	0.39	−0.33,0.31	−0.023
Monocytes (%)	23	1.93	0.46	10	1.97	0.40	−0.37,0.29	−0.090
mDC (%)	23	−1.68	0.43	10	−1.37	0.81	−0.90,0.29	−0.55
pDC (%)	23	−1.95	0.50	10	−1.69	0.92	−0.93,0.41	−0.40

TLR Expression on PBMC (MFI)								
Monocytes TLR2	23	2.75	1.33	10	2.69	1.29	−0.98,1.09	0.046
Monocytes TLR4	23	2.81	1.15	10	2.82	1.14	−0.91,0.91	−0.009
B cell TLR2	23	1.84	1.28	10	1.54	1.10	−0.62,1.21	0.24
B cell TLR4	23	1.58	1.08	10	1.62	0.90	−0.80,0.71	−0.038
mDC TLR2	23	2.13	1.00	10	1.60	1.26	−0.43,1.50	0.49
mDC TLR4	23	1.42	1.08	10	1.52	1.14	−1.00,0.80	−0.09
pDC TLR2	23	2.29	1.26	10	2.12	0.64	−0.51,0.85	0.15
pDC TLR4	23	1.87	1.08	10	2.35	0.87	−1.22,0.26	−0.47

Cytokine production by Stimulated PBMC								
GMCSF	23	2.82	1.47	10	3.66	2.19	−2.48,0.80	−0.49
IFN	23	8.24	2.18	10	8.42	2.08	−1.86,1.50	−0.084
IL10	23	0.18	1.31	10	0.31	1.76	−1.47,1.21	−0.089
IL12	23	0.22	1.13	10	0.58	1.54	−1.52,0.80	−0.29
IL1β	23	2.35	1.92	10	3.60	2.43	−3.12,0.66	−0.60
IL2	23	5.83	2.23	10	6.46	2.99	−2.90,1.63	−0.25
IL6	23	1.67	1.83	10	2.26	2.30	−2.35,1.18	−0.30
IL8	23	7.37	1.81	10	7.47	1.28	−1.23,1.06	−0.054
TNFα	23	4.92	1.58	10	5.42	2.41	−2.30,1.31	−0.27

* PBMC (1×10^6^) stimulated by PMA (50 ng/ml) and inomycin (1 µg/ml) for 16 hours.

** 95% confidence intervals (CI) are for mean differences between the two groups.

### Fecal calprotectin

**Figure 4:**
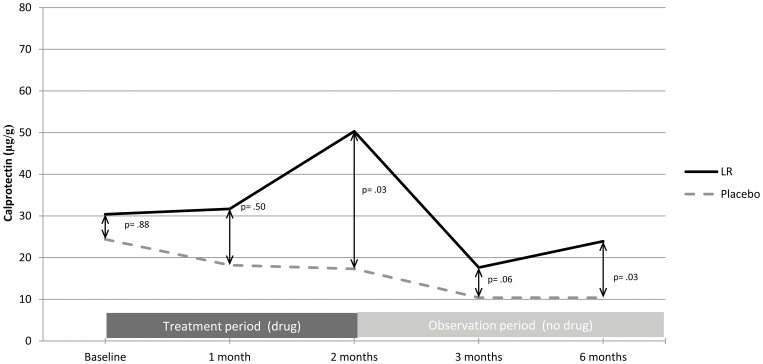
Fecal calprotectin at various study points. Median fecal calprotectin at various study time points are shown. There is a significant difference between calprotectin in LR-treated vs. placebo groups after treatment. Nonetheless all calprotectin values remained in the normal clinical range (fecal calprotectin <160 µg/g).

Stool samples were obtained at the baseline visit, the 1 month and 2 month treatment visits, as well as the 3-and 6-month observation period visits (Figure 1). There was no difference in fecal calprotectin measurements between treatment arms at baseline. The probiotic-treated group had a significantly higher fecal calprotectin level than the placebo group after 2 months of treatment: 50 µg/g (IQR 24–127 µg/g) vs. 17 µg/g (IQR 11–26 µg/g), p = 0.03 (Figure 4), although values remained in the normal clinical range (0–162.9 µg/g). Calprotectin levels returned to baseline at the 3- and 6-month observation period visits but remained modestly higher than levels in placebo-treated volunteers at these additional 2 time points (p<0.05).

### Cytokine expression by stimulated PBMC

Blood samples obtained for quantification of cytokine expression by stimulated PBMC were taken at baseline, end of 2 months of treatment, and the 6 month observation period visit. There were no differences noted comparing any of the cytokine levels evaluated in the probiotic treated or placebo groups (Table 6). We noted that in 10 patients with the highest rise of fecal calprotectin (>30 µg/g), pro-inflammatory cytokine output after 2 months of LR treatment decreased multi-fold compared to baseline for most patients (example, 8/10 showed a decrease in level of TNF-α (median decrease 4-fold), 9/10 showed a decrease in level of IL-2 (median decrease 4-fold), and 7/10 had a decrease in IFN-γ (median decrease 22-fold). However, in the patients showing a less than 30 µg/g increase in fecal calprotectin while taking LR, only 4/13 had a decrease in TNF-αproduction, only 3/13 had a decrease in IL-2 production, and only 4/13 had a decrease in IFN-γ production.

## Discussion

The current study evaluated the safety and tolerability of the probiotic bacteria strain, L. reuteri 17938 in healthy adult volunteers. Administration of the dose of 5×10^8^ CFU *L. reuteri* was well tolerated during a total of 1,952 patient days on treatment. There were no significant or severe adverse events. The adverse events that were reported were mild-moderate in severity and were not thought to be related to the study product (examples include nasal congestion, headache, vomiting). There were no clinically relevant changes on physical exam or standard blood tests.

Fecal samples were obtained at three visits during the treatment period and both observation visits (5 stool samples in total; Figure 2). We observed only marginally significant increased levels of LR in stools by qPCR in the LR treated subjects after 1 month of therapy (p = 0.06) and at the end of treatment (p = 0.08). Thus, low numbers of LR were found in the stools of these volunteers; and the probiotic was also found intermittently in some of the control stools. *L. reuteri* is known to colonize the stomach and upper small intestine [4]. It is possible that the number of viable LR were reduced during passage through the well-colonized adult colon. The low recovery of *L. reuteri* from the stool could also be explained by the low daily dose. However, the prescribed dose (10^8^ CFUs) has shown evidence of colonization as measured by fecal plating in healthy adults [4], [21]. This dose (2×10^8^ CFU) prevented diarrhea in hospitalized adults [22]. When we analyzed our cytokine and DGGE data looking only at patients in whom qPCR was positive for LR, the findings were not changed. Our studies would be enhanced by investigation of metagenomic communities, to move from structure to functional analyses in a more integrated systems biology approach. Nevertheless, we found that major shifts in the composition of fecal microbiota over time were no more prevalent in the LR- treated subjects that in controls. It is of interest that the microbial composition of many of the participants' stools (in both groups) did appear to change over time, and that the change was independent of LR ingestion.

Our data do not rule out successful colonization by *L. reuteri.* In fact, ingestion was significantly associated with a mild, reproducible increase in fecal calprotectin level. Calprotectin is a calcium dependent protein derived predominately from neutrophils, making up about 60% of cytosolic protein in human neutrophils. Therefore, the concentration of fecal calprotectin is an indication of neutrophil influx into the intestinal tract. The elevation in fecal calprotectin seen in the *LR-*treated volunteers, while unexpected, may fit with the recognized role of probiotics in augmenting antimicrobial peptide secretion in the intestine. For example, humans who consume any one of several probiotic *E. coli* strains have 8–10-fold increase in intestinal human beta-defensin (hBD-2) secretion by Paneth cells [23]. Calprotectin secreted by neutrophils has a demonstrated antimicrobial effect, aiding in the killing of fungi and Staphylococci by sequestration of nutrient metals, such as zinc and manganese [24]. We suggest that either the LR itself or a bacterial product may produce a mild increase in mucosal inflammation, although we stress that the fecal calprotectin levels remained well within the normal range in all volunteers.

The indigenous microbiota maintains a constant interaction with the host immune system. Recent studies have suggested that various immune populations may be regulated by the microbiota. While this study was not powered to evaluate changes in immune parameters, we were able to describe the findings of various immune related activities in healthy adults before and after treatment with LR. There were no differences in circulating composition of peripheral blood mononuclear cells (PBMC), including Tregs, comparing the LR-treated and the placebo-treated groups. Previous studies have generally found that probiotic administration in adult humans do not significantly impact general immune parameters, such as natural killer cell activity, phagocytic and respiratory burst activity, serum immune globulin levels, or *in vitro* PBMC cytokine release in response to lipopolysaccharide [25]. Local gut immune responses likely do not parallel observations made using peripheral blood mononuclear cells. In a rat model, we are able to detect the effect of LR on local gut immune response. We have shown that feeding LR in neonatal rats changed T cell subsets in the intestinal mucosa (DDW 2012 annual meeting, abstract#Su1938) and inhibited proinflammatory cytokine production in the gut [15]. It is not realistic to use intestinal tissues to study local gut immune responses in normal adult volunteers. Recent studies demonstrated that intraepithelial lymphocytes may consist of CX3CR1+ PBMCs migrating from the peripheral blood into the gut epithelium [26] We intended to identify whether administration of *L. reuteri* at the studied dose would trigger the patient's PBMCs to respond to the stimulants in vitro.

In several studies, interleukin-10 and −12 levels were enhanced by probiotics [25]. In a large adult study of probiotic treatment for irritable bowel syndrome (*L. salivarius* UCC4331 or *Bifidobacterium infantis* 35624), the authors performed *in vitro* PBMC stimulation assays. However, in that study, the only reported effect of the probiotic treatment was that *B. infantis* reduced the IL-10 to IL-12 ratio, indicating inhibition of the Th-1 pro-inflammatory state [27]. In our studies, there was not significant change in expression of cytokines, including IL-10 and IL-12p70 by stimulated PBMC. However, there was a trend toward significance in decreased IL-1β expression in the probiotic treated group (p = 0.09). Our ratios of IL-10 to IL-12p70 were approximately 1∶1 at baseline, compared to 50∶1 at baseline in the study of O'Mahony *et al.* [27]. This discrepancy could be caused by one or more differences in our experimental protocol. Our studies used media that was serum free and specific for human PBMC, X-VIVO-15 [28], [29]. This is in comparison to 10% fetal calf serum in the cited study [27]. In addition, we used a 24 hour time to collect cytokines compared to 72 hours in the mentioned study. There may be intrinsic differences in IL-12p40 level measured [27] and IL-12p70 that was measured in our study. Nevertheless, we found no augmentation of the ratio of IL-10 to IL-12 (baseline ratio = 1.7; ratio after 56 days of LR = 0.8), compared to a 2-fold increase in their studies. This discrepancy could be related to the above technical considerations or to the probiotic chosen (*L. salivarius* versus *L. reuteri*).

Multiple studies have compared various standard-sensitivity multiplex cytokine assays [30], [31] or enzyme-linked immunosorbent assay (ELISA) [32] (used by O' Mahony *et al*), flow cytometry cytokine bead arrays [33], or electrochemiluminescent Meso Scale Discovery (MSD) assays [34] (used in our study). These studies have shown variable agreement among assays and have indicated that absolute cytokine concentrations differ across testing platforms, even though commercialized available assays include the calibrators generally developed by using WHO cytokine references [35]. Such standards are usually calibrated in International Units which are arbitrarily defined by bioassays in standard cell lines [36]. There are differences between measuring the concentrations of cytokines in human plasma with biological activities standardized by WHO and the measurement of cytokine output *in vitro* by PBMC. It has been noted that in a study of multi-analyte bead-based (Luminex) kits, WHO cytokine standards were assayed at the same expected concentrations as the standards provided with each kit, but WHO and kit standards often yielded very different absolute concentrations [37]. Therefore, we suggest that studies of probiotics should use longitudinal measurements in which relative, rather than absolute, levels in cytokines are measured [38].

As mentioned, a post-hoc analysis was performed focused on the 10 patients in the probiotic treated group who had elevations in fecal calprotectin >30 µg/g from baseline to end of treatment; those with the largest increase in fecal calprotectin had largest reduction in cytokine output. While this is merely a description of our findings and by no means a claim of linkage, an interesting idea for future studies may be to investigate the role of intestinal inflammation in the regulation of anti-inflammatory pathways.

Mouse models have indicated LR treatment attenuates inflammatory processes [39] by a mechanism characterized at least in part by the induction of Tregs. We anticipated a mild but measurable effect of LR treatment on circulating Treg numbers. However, in normal adults with a well-established microbiota, such a response was not demonstrated. Treg responses to probiotic (*B. infantis* 356240) have been observed in mouse models of inflammation, but they were observed in isolated spleen cells, not in circulating blood mononuclear cells [40]. Our study will hopefully facilitate future studies of Tregs in younger, more immunologically naive populations using *L. reuteri* and other probiotics.

It is important to note that intention to treat (ITT) analysis is a conservative method and may have slightly underestimated potential side effects in this safety evaluation. We compared the incidence rates of potential adverse events between the two study arms for the total number of days (560 days, assuming 100% compliance) that volunteers took placebo and compared this value to that of the total number of days that the 30 participants in the treatment arm (total number of days on treatment = 1680) took LR. However, due to less than 100% compliance in the treatment arm, there were only 1500 patient days on treatment; so we may have underestimated the incidence of AEs in the treatment arm by 11%. This implies that per protocol (PP) analysis would have resulted in 11% higher incidence rates for adverse events.

This safety study, conducted in a rigorous randomized controlled manner, demonstrates that Lactobacillus reuteri DSM 17938 is safe and well tolerated in healthy adults. From a practical standpoint, not only does this offer important clinical safety information, but may pave the way for future therapeutic trials of this strain of probiotic to be conducted with FDA oversight to allow for appropriate product labeling and fit for intended purpose use. Future trials should also investigate if immune responses such as an increase in Tregs and changes in fecal calprotectin might be observed in hosts with a less diverse microbial population and with an incompletely developed immune system, such as in newborn infants with colic.

## Supporting Information

Protocol S1 **Study protocol.**
(PDF)

Checklist S1 **CONSORT checklist.**
(DOC)

Materials and Methods S1 Detailed materials and methods for measuring the expression of TLRs, percentage of Tregs in PBMC by flow cytometric analysis, cytokine production by stimulated PBMC, fecal calprotectin, and microbiota analysis.
(DOCX)
