# Supplementary material for: Safety and Tolerability of Lactobacillus reuteri DSM 17938 and Effects on Biomarkers in Healthy Adults: Results from a Randomized Masked Trial
Source: PLoS One. 2012 Sep 6;7(9):e43910. doi: 10.1371/journal.pone.0043910 (PMC3435331; doi:10.1371/journal.pone.0043910)
Supplement: Protocol S1 — Study protocol. (PDF) [file pone.0043910.s001.pdf]

1.0 General Information

1.1 *Please enter the full title of your study:

Phase I Safety and Tolerance of Lactobacillus reuteri in Adults

1.2 *Please enter the Study Alias you would like to use to reference the study:

Safety and Tolerance of Lactobacillus reuteri in Adults

2.0 Add Department(s)

2.1 List of Departments associated with this study:

Primary Dept?	Department Name
◉	**UT-H** - *MS* - Pediatrics-Gastroenterology

3.0 Assign key study personnel(KSP) access to the study

3.1 *Please add a Principal Investigator for the study:

Rhoads, J Marc, MD

Select if applicable

☐ Student

If the Principal Investigator is a Student, the name of the Faculty Advisor must be supplied below.

3.2 If applicable, please select the Protocol Staff personnel:

A) Additional Investigators

Tran, Dat, MD
Co-Investigator

B) Research Support Staff

Fatheree, Nicole - Study Coordinator
Mangalat, Nisha - Research Associate

3.3 *Please add a Study Contact:

1. Fatheree, Nicole
2. Rhoads, J Marc, MD

The Study Contact(s) will receive all important system notifications along with the Principal Investigator. (e.g. The study contact(s) are typically either the Study Coordinator or the Principal Investigator themselves).

3.4 If applicable, please add a Faculty Advisor:

No Faculty Advisors have been added.

3.5 If applicable, please select the Designated Department Approval(s):

1. Colasurdo, Giuseppe
   Dean

3.6 If applicable, please select the Administrative Assistant(s)

1. Ramos, Susan

Administrative Assistants have READ-ONLY access to submissions in iRIS.

4.0 Contact Information

4.1 * Primary Contact (PI or Study Coordinator):

J. Marc Rhoads

4.2 * Phone Number:

713-500-7642

4.3 * Evening Phone Number:

713-208-1732

4.4 Pager Number:

713 509 4830

4.5 Secondary Contact (PI or Study Coordinator):

Nicole Fatheree

4.6 **Secondary Contact Phone Number:**

713-500-5669

4.7 **Secondary Contact Evening Phone Number:**

713-502-3911

4.8 **Secondary Contact Pager Number:**

713-509-0463

4.9 * **Please list the names and credentials and ROLES for all Principal Investigators and Co-Investigators on this study.**
(Examples include John Smith, PhD - Statistician; Mary Jones, MD - Surgeon; Jane Doe, MD - Patient Recruitment)

J. Marc Rhoads, M.D. - Principal Investigator

5.0 Additional Study Personnel

5.1 **List any additional "key study personnel" not listed in the UT directory and their role on this study.**

Key study personnel are defined as personnel responsible for the design, conduct, or reporting of the proposed research or other educational activities.

* **NOTE: Human subjects training certification and research conflict of interest forms are required for these individuals.**

NA

5.2 **List all additional personnel not considered "key study personnel" and their role on this study.**

Key study personnel are defined as personnel responsible for the design, conduct, or reporting of the proposed research or other educational activities.

* **NOTE: Human subjects training certifications are required for these individuals.**

Dat Tran, MD. He will advise on the in vitro studies of regulatory T-cells and circulating dendritic cells.

6.0 Locations

6.1 * **Is the research being conducted at LBJ Hospital?**

○ Yes ● No

6.2 * **Is the research being conducted at Memorial Hermann Healthcare System?**

Please note: TIRR is now part of the Memorial Hermann Healthcare System.

(•) Yes ( ) No

6.3 *** Is the research being conducted at the Clinical Research Unit (CRU)? The CRU was formally known as the University Clinical Research Center (UCRC).**

NOTE: If YES, you will be required to complete some additional questions within this application.

(•) Yes ( ) No

6.4 **Please identify other locations or facilities where the research is being conducted:**

Select all that apply:

- [ ] Ben Taub General Hospital
- [ ] HCPC - Harris County Psychiatric Center
- [ ] HISD - Houston Independent School District
- [ ] Houston Medical Center Building
- [ ] M. D. Anderson
- [ ] Methodist Hospital
- [ ] MSI - Mental Sciences Institute
- [ ] St. Luke's Episcopal Hospital
- [ ] Texas Childrens' Hospital
- [ ] Texas Heart Institute
- [ ] Thomas Street Clinic
- [ ] UT - Dental Branch
- [x] UT Professional Building (formally Hermann Professional Building)
- [ ] UT - School of Nursing
- [ ] UT - School of Public Health
- [ ] Valley Baptist Medical Center - Brownsville (VBMC)
- [ ] Veterans Affairs Medical Center
- [ ] OTHER

6.5

Please identify additional locations or facilities not listed above:

NA

If using other facilities, do they require additional IRB review?

( ) Yes ( ) No

What is the status of this other IRB review?

( ) Not yet submitted
( ) Pending
( ) Approved

If necessary, provide additional information here:

NA

7.0 Memorial Hermann Hospital Locations

7.1 Select all locations that apply:

Please note: If conducting research at any of the Memorial Hospital Healthcare locations, the Memorial Hermann Healthcare Research Application must also be completed and attached to your submission packet.

- [ ] Memorial Hermann Hospital, Houston, TX
- [ ] Memorial Hermann Children's Hospital, Houston, TX
- [ ] Memorial Hermann Fort Bend Hospital, Missouri CIty, TX
- [ ] Memorial Hermann Memorial City Hospital, Houston, TX
- [ ] Memorial Hermann Southeast Hospital, Houston, TX
- [ ] Memorial Hermann The Woodlands Hospital, The Woodlands, TX
- [ ] Memorial Hermann Katy Hospital, Houston, TX
- [ ] Memorial Hermann Southwest Hospital, Houston, TX
- [ ] Memorial Hermann Northwest Hospital, Houston, TX
- [ ] Memorial Hermann University Place Hospital, Houston, TX
- [ ] Memorial Hermann Continuing Care Hospital, Houston, TX
- [ ] Memorial Hermann Corporate Office
- [ ] The Institute for Rehabilitation and Research (TIRR)
- [ ] TIRR Rehabilitation Centers (TRC)
- [x] CRU use outside Memorial Hermann Hospital System facilities and services

8.0 Clinical Research Unit (CRU)

8.1 * Type of Project:

CRU Category

- (•) A (Investigator initiated)
- ( ) B (Patient hospitalized)
- ( ) D (Industry sponsored)

8.2 * Justification for Category Selection above:

For Category A studies with any support from industry, explain the process of the protocol's initiation and subsequent implementation. Explain the extent of industry support for the study.

The protocol was initiated by the PI in response to an FDA requirement of a pending IND for the use of Lactobacillus reuteri in babies with colic. This protocol is to demonstrate the safety and tolerability of Lactobacillus reuteri at ascending doses in adults. The FDA has suggested that the infant study will likely be evaluated favorably if the Phase I study in adults is successful.

8.3 * Justification for use of CRU:

Explain why the use of the CRU is desirable or necessary for the performance of the protocol. This may include the need for research nursing expertise, physical facilities, ancillary support, etc.

The CRU is a safe, organized, and patient-friendly facility. The CRU is accessable to patients as well as researchers. This study involves blood drawing and a complete physical examination on 4 occasions is required.

8.4 Estimated Duration of Project to be done in the CRU:

(e.g. How long is the clinical component of the study?)

6 months

8.5 Estimated start date:

05/05/2008

8.6 Number of patients to be admitted simultaneously (if applicable):

1

8.7 * Who will be managing your data?

- (●) The PI (Principal Investigator)
- ( ) CRU (Clinical Research Unit)

If you answered the CRU, please provide your data sharing plan. Include your expected schedule for sharing data, the methods to reduce the risk for subjects identification, the acceptable methods of data sharing, availability of data documentation, whether a data sharing agreement will be required, the mode of data sharing and the format of the final dataset.

8.8 CRU Services Requested:

Select all that apply:

- [ ] Clinical research nursing - inpatient
- [x] Clinical research nursing - outpatient
- [ ] Clinical research nursing - off-site
- [x] Study coordinator support
- [ ] Ancillaries (i.e. diagnostics, pharmacy)
- [ ] Informatics (i.e. data mgmt) + biostatistics
- [ ] Informatics only (i.e. data management)
- [ ] Bionutritionist
- [x] Clinical space
- [ ] Other

If selecting "other", please provide a description:

9.0 Screening Questions for Exempt Research

9.1 Does the research involve normal educational practices that are conducted in commonly accepted educational settings?

( ) Yes (●) No

9.2 Does the research involve use of educational tests, surveys, interviews, or observations of

public behavior?

○ Yes ◉ No

9.3 Does the research involve the collection or study of already existing data, documents, records, pathological specimens, or diagnostic specimens?

○ Yes ◉ No

9.4 Does the research evaluate public benefit or service programs?

○ Yes ◉ No

9.5 Does the research evaluate the taste and/or quality of food?

○ Yes ◉ No

9.6

If you answered "YES" to any of these questions, your research MAY be exempt from review by the CPHS. To further consider this possibility, click on the HELP LINK to the right to review the information from the Code of Federal Regulations.

10.0 Screening for Expedited Review

10.1

The Committee for the Protection of Human Subjects employs a review process that conforms to Federal regulations (both FDA and OPRR/HHS) and is supplemented by institutional regulations. The review process is the same for all research involving human subjects or human derived materials and data whether funded or non-funded.

Primary regulatory codes are in "Federal Policy for Protection of Human Subjects: Common Rule" (HHS), 45 CFR 46 and Food & Drug Administration 21 CFR 50 and 21 CFR 56. The type of review that a study receives depends on the risks posed to potential subjects by the research. These risks include the probability and severity of possible harm to the subjects' physical, psychological, social, legal or economic welfare. Minimal risk research activities in which the only involvement of human subjects is in one or more the categories noted below may qualify for expedited review by the CPHS.

To assist the Office of Research Support Committees assess the appropriate type of review necessary for your research proposal, please answer the following question.

10.2 * Does your research protocol qualify for "expedited" review?

For more information on "expedited" review categories, please click on the HELP LINK to the right of this panel.

○ Yes

◉ No or Not Sure

11.0 Funding Source

11.1 * Have you applied for outside funding?

○ Yes ◉ No

11.2 * **Has this study undergone scientific peer-review by one of the following institutions or agencies: M. D. Anderson Cancer Center, National Institutes of Health (NIH), Center for Disease Control (CDC), Department of Defense (DOD), National Institute of Justice (NIJ) or American Heart Association (AHA) ?**

○ Yes ◉ No

11.3 **Granting Agency/Sponsor (You can select more than one agency.):**

	Sponsor	Funding
**Department**		
**Federal - NIH**	NIH/specific Institute or Center unknown	☑
**Private - Non-profit**		
**Academic Health Center**		
**Other**		
**Federal**		
**Internal - UTHSC-H**		
**Pharmaceutical or Device Manufacturer**		
**State Agency**		

11.4 **Status of funding:**

Please select one:

○ Applied/Pending
○ Approved
◉ Not Applicable

11.5 **Are part of or all activities in this proposal funded by a training grant?**

(●) Yes ( ) No

12.0 Purpose and Objectives

12.1 * Has this study previously been reviewed by CPHS and withdrawn or rejected?

( ) Yes (●) No

If YES, please indicate the date and number if available:

12.2 * Research Summary
(500 words or less)

The summary required for this section of the application should include the following information. Please note that this summary must be in lay language, as this summary is reviewed by all members of the CPHS, including our non-technical/non-medical members. Summaries not provided in lay language will be sent back for revision, which may possibly result in a delay of the protocol's review.

In your summary, please use these **bold** headings.

- **Purpose**
- **Procedures**
- **Course of Study**
- **Enrollment**
- **Recruitment**
- **Known Risks**
- **Data Safety Monitoring**
- **IND#**
- **Proposed Funding Source**
- **Communication of Study Results**

For more information, please refer to the HELP BUBBLE on the right side of this panel.

System will timeout after 40 minutes - Please **SAVE YOUR WORK OFTEN** by pressing the **OK button** within the text editor.

Purpose:
To demonstrate that Lactobacillus reuteri DSM 17398 is a safe and well tolerated in adults. The development of this product under FDA guidance will allow for the product labeling and quality control of this biologic . It is now marketed only as a nutritional supplement. If shown to be efficacious in phase II/III trials, we believe this will allow for the greater use and acceptance of this product as a viable treatment for infants with colic.

We hypothesize that these healthy adults will not experience any significant symptoms.

Procedure and course of study:
Patients will be randomized to 3 treatment groups and 3 placebo groups in an ascending dosage model. The first Arm of the cohort will include 6 patients on LR and 2 on placebo. The 6 treatment patients will receive *LR* at 5 drops (0.2 ml, 10^8 c.f.u.) twice daily during the first two months. The second arm of the cohort will start treatment during the second month. The second cohort will include 6 patients on LR and 2 on placebo. The 6 treatment patients will

receive *LR* at 10 drops (0.4 ml, 2 x 10^8 c.f.u.) twice daily during the first two months. The third arm of the cohort will start treatment during the third month. The third cohort will include 6 patients on LR and 2 on placebo. The 6 treatment patients will receive LR at 15 drops (0.6 ml, 3 x 10^8 c.f.u.) twice daily in the third month.

Placebo will be the equivalent number of drops of suspended sunflower oil (without LR), provided by Biogaia.

This trial will show safety and tolerability of LR at escalating doses. The treatment group will be given LR for a 2-month period, followed for a 6-month observation period. Arms 1, 2 & 3 will start a month apart, allowing us to monitor safety of lower dosages before higher doses.

Enrollment:
The project will be announced at a departmental meeting and advertised by Flyer throughout the medical school. A consent form, approved by the institutional review board of University of Texas Health Sciences Center at Houston will be provided to all who are interested. The participants or their parents and guardians will be encouraged to ask questions regarding the study and why they are being asked to participate in it.

On screening day (day 1) subjects will be evaluated by a physician to obtain informed consent and in order to determine if the potential subject meets all enrollment criteria. Basic demographic data and clinical data, including use of medications and use of herbal or homeopathic treatments, will be recorded. Subjects who meet inclusion criteria shown above and who do not meet exclusion criteria above will have blood collected to test for HIV serum antibody, Hepatitis B surface antigen, and Hepatitis C antibody. Urine pregnancy testing will be done for all age-bearing female subjects. These tests will be preformed in our laboratory and reported as promptly as possible, usually within 48 hours, to determine if participants meet exclusion criterion. Subjects with negative results on these HIV and Hepatitis diagnostic tests will progress to phase 2 of screening.

Risks:
Fever: Fever is defined as oral temperature $\geq$ 39.0 degrees C
Diarrhea: Diarrhea is defined as three loose bowel movements in a 24-hour period. The episode will be defined as concluding on the first day without three loose bowel movements. However, if a subject has three or more loose bowel movements within a 3-day period following the end of an episode, that day and the intervening day(s) will be considered as part of same episode.

Regarding the safety and tolerance of the probiotic strains of Lactobacilli, there have been rare reports of Lactobacillus bacteremia, meningitis, fungemia, endocarditis, and D-lactic acidosis (1, 2). Some probiotics, Lactobacillus GG and LR, are known to exist naturally in the human flora and are linked to normal health in the digestive system. Seven articles were found to have reported complications with probiotics, mainly in a very small group of adults, starting in the early 1980s from P. M. Sherman, personal communication, 09/2007. Four cases occurred in infants. These are described in the Protocol.

Data Safety Monitoring:
A DSM has been established for the newborn colic study, consisting of Drs. Gloria Heresi, Pediatric Infectious Diseases, Dr. Ruben Quiros, Pediatric Gastroenterology, and Mike Fant, Neonatology. For the Phase 1 study, we will add Dr. Herbert Dupont, Medicine Infectious Diseases, so that thePhase1 committee will include Drs. Heresi, Quiros, Fant, and Dupont. This committee will meet monthly for the first 3 months and at the end of the study.

IND#: 13561

Funding source: PI has word from NIH-NCCAM on 4/10/08 that $40,000 will be allocated for this part of the project.

Communication of study results: The PI will communicate these results to the FDA and NCCAM. The results will not be published, unless unforeseen adverse events are observed.

13.0 Study/Population

13.1 * Age

Select all that apply:

- [ ] 0-6 (parental consent only, Pediatric Assessment required)
- [ ] 7-11 (Requires child's assent plus parental permission, Pediatric Assessment required)
- [ ] 12-17 (Requires consent plus parental permission, Pediatric Assessment required)
- [x] 18+ (Requires consent only)

Enter the specific age range for study population (if overlap or specific within a category):

19.00
to
60.00

- ( ) months
- (•) years

13.2 *Gender

- ( ) Male
- ( ) Female
- (•) Both male and female

13.3 * Is this a multi-center trial?

( ) Yes (•) No

13.4 * How many subjects do you need to complete the study?

40

*** To achieve that number, how many subjects do you need to enroll/consent at this institution/site?**

65

If necessary, provide explanation below:

13.5 * **What is the number of participants that will be enrolled at all sites?**

24

If necessary, provide explanation below:

13.6 * **Justification for the number of subjects required:**

Regarding safety and tolerance of a drug, Lactobacillus reuteri, 24 adults over a 6 months period will show, if any, diversity in adverse effects.

13.7 **Please designate if any vulnerable populations will be included in the study.**

Check all vulnerable populations that are included:

- [ ] Children
- [ ] Pregnant Women
- [ ] Physically disabled
- [ ] Mentally disabled
- [ ] Economically or educationally disadvantaged persons
- [ ] Prisoners
- [ ] Other

If other, please explain below:

13.8 **Identify Inclusion criteria:**
NOTE: Submission will be returned if inclusion criteria is not complete.

If you want to list the criteria one statement at a time, select the link to "add inclusion criteria". You will be prompted to enter the criteria as individual statements. This information can then be used during the screening process for recruitment of subjects. **If you do not want to enter the information in this fashion, select the link to access the text editor.**

No Inclusion criteria has been associated.

Healthy Adults (19 to 60 years old) with no other recognized illness will be enrolled. There will be no selection on the basis of age, race, or gender. Females of childbearing potential are allowed but will be required to agree to practice one of several types of double-barrier methods of birth control ; currently pregnant will be excluded from the study. A pregnancy test (urine) will be performed on females participating in the study (at each visit).

13.9 **Identify Exclusion criteria:**
NOTE: Submission will be returned if exclusion criteria is not complete.

If you want to list the criteria one statement at a time, select the link to "add exclusion criteria". You will be prompted to enter the criteria as individual statements. This information can then be used during the screening process for recruitment of subjects. **If you do not want to enter the information in this fashion, select the link to access the text editor.**

No Exclusion criteria has been associated.

Pregnancy or breastfeeding

Patient taking immunosuppressive medications, including oral corticosteroids
Positive result of HIV, Hep B, and/or Hep C test
Gastrointestinal related diseases and surgeries
No more than two study participants in one household
Use of probiotics in the last 90 days
Diarrheal illness within the past 30 days
Recent or current use of oral antibiotics /anti-fungals(in the past 2 weeks)
Current use of oral laxatives
Chronic alcohol use or more than 1 drink per day
Known sensitivity to safflower oil or products containing linolenic/oleic acids
Will require that subject not take any other probiotic-containing products, including yougurt supplemented with probiotics during the 6-month period.

13.10 * Is any racial/ethnic group excluded?

○ Yes ◉ No

13.11 Provide justification for inclusion or exclusion of any group (gender, race, or other):

N/A for this study.

14.0 Targeted/Planned Enrollment Table

Please complete the following table with the number of subjects in each category:

Ethnic Category	Females	Males	Total
**Hispanic or Latino:**	4	4	8
**Not Hispanic or Latino:**	8	8	16
**Ethnic Category: Total of all Subjects:**	12	12	24

Racial Categories	Females	Males	Total
**American Indian/Alaska Native:**	0	1	1
**Asian:**	2	1	3
**Native Hawaiian or Other Pacific Islander:**	2	2	4
**Black or African American:**	2	2	4
**White:**	6	6	12
**Racial Categories: Total of All Subjects:**	12	12	24

15.0 Use of Study Drugs

15.1 * Will drugs be used in this study?

(•) Yes ( ) No

16.0 Study Drugs Details

16.1 If YES, is this study being conducted under an approved IND?

( ) Yes (•) No

16.2 * List the study drugs used:

Drug List

Trade Drug Name:	Lactobacillus Reuteri
Generic Drug Name:	Lactobacillus Reuteri
Investigational Drug Name:	
Manufacturer name of drug:	Biogaia
Is the drug supplied at no cost?	Yes
FDA Approved:	No
A new drug or a new use of approved drug:	No
IND Necessary:	No
IND Number:	13216
Frequency:	Twice a day for 2 months
Possible Untoward Effects, Their Symptoms & Treatment:	
Investigators Authorized to Prescribe:	

17.0 Medical Devices

17.1 * Will medical devices be used in this study?

( ) Yes (•) No

18.0 Methodology

18.1 * Study Type - Check all that apply:

- [ ] Therapy/Intervention/Prevention
- [x] Pilot or Feasibility Study
- [ ] Diagnosis or Diagnostic Test
- [ ] Etiology/Harm/Risk Factors (including Molecular Genetics)/Mechanisms of Disease
- [ ] Prognosis
- [ ] Descriptive or Prevalence
- [ ] Prospective
- [ ] Retrospective
- [ ] Other

If "other" or more description necessary, enter information below:

18.2 * Is this a research project that prospectively assigns human subjects to intervention and comparison groups to study the cause and effect relationship between a medical intervention and a health outcome or is this a pediatric post-market surveillance device study?

( ) Yes (•) No

18.3 * Please select from the following options:

(•) My study is investigator initiated (e.g. are you the lead sponsor or sponsor-designee?)
( ) My study is industry or NIH sponsored.

If the answer to the previous question is "Yes" and this study is investigator initiated, you are required to register your study at http://register.clinicaltrials.gov before enrollment begins or no later than 21 days after the first patient is enrolled and update every 12 months. For more information, please visit http://www.uth.tmc.edu/research/clinical/ctregistration.html, and/or contact Gena Monroe at Gena.Monroe@uth.tmc.edu

If your study is industry or NIH sponsored, the sponsor is required to register your study. Please confirm with the sponsor that your study has been registered at http://clinicaltrials.gov , and file the registration number with your study documents.

18.4 * Describe experience of subjects while participating in this research.

Participant will ingest treatment of Lactobacillus reuteri during a two month period at ascending doses. Safety and tolerance will be observed over a six month period.

18.5 Specify which procedures and/or diagnostic tests are considered routine clinical care.

Physical Examination

18.6 Specify which procedures and/or diagnostic tests are being performed for research purposes only.

Electrolyte Testing

Are these tests being paid for by the sponsor?

(•) Yes ( ) No

If not, please explain:

18.7 Specify which of the research procedures and/or diagnostic tests are being performed solely to screen for study eligibility.

Pregnancy Test will be administered prior to enrolling into the study.

Are these being paid for by the sponsor?

(•) Yes ( ) No

If not, please explain:

18.8 * Will interviews, questionnaires and/or surveys be part of the study?

(•) Yes ( ) No

18.9 * Describe method of data analysis:

Study coordinator has secured database on clinical server only accessable to PI and study coordinator.

18.10 Summarize procedures to protect the confidentiality and anonymity of subjects:

PI and study coordinator have access to database. Data is deidentified once inserted into database.

19.0 Risks & Benefits

19.1 * Describe the plan for monitoring data and safety.

Include: Frequency of monitoring, description of stopping rules for individual patient and overall study and frequency of adverse event reporting to sponsor if applicable.

Data and Safety will be monitored every two weeks during treatment of Lactobacillus reuteri thereafter once every month by telephone for long term adverse effects. The first two months will require the patient to make a descriptive account of, if any, adverse effects by populated a diary and a bi-weekly questionnaire. The months following the treatment will require the participant to complete a telephone survey. If any severe adverse effects are reported such as a fever extending over a three day period the study must be stopped.

19.2 * Describe the possible risks to participants (including psychological harm, economic harm, social stigmatization, legal harm and physical harm if applicable). Include justification of those known risks.

Possible effects include vomiting, fever, flatulence, and diarrhea.

19.3 Describe ways in which this risk, if any, will be minimized. Include tests, evaluations, and/or observations that will be used.

Risk are at a minimum because of the ascending dose scheduling implemented in the study design. Moderate sized studies in the European literature attest to the safety of this product.

19.4 Is there potential for direct benefit to the subject?

○ Yes ◉ No

19.5 Will there be benefit to the class of subjects ?

○ Yes ◉ No

19.6 Is there societal benefit?

◉ Yes ○ No

19.7 - If yes to any of the last three questions, explain:

The benefits of dietary supplementation of healthy adults are unclear, but we have postulated that there be a major impact on colic symptoms (crying time) and breath hydrogen of probiotics in babies with colic. There is good evidence that probiotics reduce diarrheal disease severity in children. During the course of a 2-month study, there is a chance of reduced gastroenteritis episodes for the subjects.

20.0 Risk Categorization

20.1 * Please check all boxes that apply to your study in each of the following groups.

Group 1 - If necessary, select the "none of the above" option.

- [x] Collection of blood samples by finger stick, heel stick, ear stick, or venipuncture as follows: from healthy, nonpregnant adults who weigh at least 110 pounds. For these subjects, the amounts drawn may not exceed 550 ml in an 8 week period and collection may not occur more frequently than 2 times per week.
- [ ] Collection of blood samples by finger stick, heel stick, ear stick, or venipuncture as follows: from other adults and children1, considering the age, weight, and health of the subjects, the collection procedure, the amount of blood to be collected, and the frequency with which it will be collected. For these subjects, the amount drawn may not exceed the lesser of 50 ml or 3 ml per kg in an 8 week period and collection may not occur more frequently than 2 times per week.
- [ ] Prospective collection of biological specimens for research purposes by noninvasive means.
- [ ] Collection of data through noninvasive procedures (not involving general anesthesia or sedation) routinely employed in clinical practice, excluding procedures involving x-rays or microwaves. Where medical devices are employed, they must be cleared/approved for marketing.
- [ ] ** None of the above

Note: For this purpose, in the state of Texas, children are defined as those persons under the age of 18.

20.2

Group 2 - If necessary, select the "none of the above" option.

- [ ] Low risk intervention in a population at risk for serious clinical events based on underlying disease.
- [ ] Intervention of undefined risk or intervention with low frequency of serious adverse events.
- [ ] Low risk studies in vulnerable populations such as pregnant women, children or prisoners.
- [ ] Questionnaires eliciting personal information that may pose risk to confidentiality.
- [ ] Interventions associated with risk of serious adverse events at high or uncertain frequency.
- [ ] Studies in populations associated with very high risk of serious adverse clinical events based on underlying disease or in whom assessment of treatment-associated adverse events may be difficult.
- [ ] Prior data suggests that the intervention being studied has the potential to induce unacceptable toxicity.
- [ ] Trial evaluates mortality or another major endpoint, such that inferiority of one treatment arm has safety as well as effectiveness implications.
- [ ] Is ethically important for the trial to stop early if the primary question addressed has been definitively answered, even if secondary questions or complete safety information were not yet fully addressed.
- [x] ** None of the above.

21.0 Recruitment and Advertising

21.1 * How will subjects be recruited? Check all that apply:

- [x] Direct contact in a medical setting
- [x] Direct contact in a non-medical setting
- [ ] Newspaper Ad
- [x] Broadcast media (television/radio/internet)
- [x] Posted Notice
- [ ] Letter
- [ ] Other

Explanation and location for any of the above:

> We will advertise by Flyers at the medical school, by Craiglist on the Internet, and by personal request outside of the hospital environment.

21.2 * Who will do the recruiting?

> Study Coordinator and PI

21.3 * Are you screening or recruiting from or through the patient base of a healthcare provider?

(●) Yes ( ) No

22.0 HIPAA - Guide to completing the appropriate forms - Screening and Recruitment Waiver

22.1 * Are all subjects recruited from the patient base of one of the research team investigators (primary investigator or co-investigator?

( ) Yes (●) No

23.0 IRB Waiver of Authorization - SCREENING AND RECRUITMENT

23.1

Based on your answers to the "HIPAA - Guide to completing the appropriate forms" questionnaire, your study requires that you complete the "**IRB Waiver or Alteration Form - Screening or Recruitment"** form. This form is appropriate for all studies that need a HIPAA waiver due to the fact that in this study, the researcher does not have direct contact or a treatment relationship with research subjects prior to recruitment and cannot obtain patients' authorization for the use and disclosure of PHI about themselves for recruiting purposes. In summary, the research cannot practicably be conducted with authorization, and it is not practiable to conduct screening and recruiting activities of subjects for this study without access to and use of the Protected Health Information (PHI).

When you are in the initial submission form of iRIS, you can select and complete this form from the "Review Board Form" section of the form.

24.0 Consent Procedure

24.1 * Describe the process of obtaining subjects' consent (Include where it will take place, who will obtain the consent and how cultural issues will be addressed, etc.)

Patient will be recruited from local clinics or referrals and scheduled a face to face interview. Obtaining consent will take place in the CRU in Memorial Hermann Hospital.

24.2 * Will non-English speaking people be approached to participate in this study?

○ Yes ● No

24.3 * Will a translation be available for non-English speaking subjects?

● Yes ○ No

24.4 If the study involves minors, describe the processs of parental permission and how the assent of the minor will be sought.

N/A

24.5 * In case of injury, please explain who will pay for the treatment.
NOTE: If not applicable, please note "N/A".

N/A

25.0 Compensation/Costs

25.1 * Will subjects receive any compensation for participation in this study?

● Yes ○ No

25.2 Describe compensation and/or remuneration being offered to subjects. Provide amount and justification.

$30 dollars including parking each visit.

25.3 * **Will there be any costs to subjects associated with their participation in research?**

◯ Yes ◉ No

If so, please explain:

26.0 Specimens and Cell Lines

26.1 * **Will Specimens be obtained specifically for this study?**

◉ Yes ◯ No

27.0 Specimens and Cell Lines Details

27.1 Will specimens be obtained from residual samples?

◯ Yes ◉ No

27.2 Will specimens be maintained in such a way that the subjects can be identified?

◉ Yes ◯ No

If so, how will confidentiality be preserved?

Specimen will be maintained in UT secure data files.

27.3 Will this study involve stem cells?

◯ Yes ◉ No

27.4 Will this study involve stored tissue samples?

◯ Yes ◉ No

27.5 Will saved samples or their derivatives have the potential to produce profits for the investigators or the university?

◯ Yes ◉ No

27.6 Does this study involve cell lines?

◯ Yes ◉ No

If Yes, will cell lines or data be obtained from a public repository or a public source?

◯ Yes ◯ No

If Yes, will these cell lines or data be linked directly to the subject from whom they were obtained?

◯ Yes ◯ No

27.7 Does this study involve the storage of genetic data in electronic form?

◯ Yes ◉ No

27.8 Does this study involve gene transfer?

○ Yes ◉ No

28.0 Institutional Safety Committees

28.1 Does your work involve the use of human blood, tissue, cells, microorganisms, or recombinant DNA?

◉ Yes ○ No

If so, please list:

Serum electrolyte testing; urine pregnancy testing.

Current Institutional Biological Safety Committee approval number:

28.2 Do you have a laboratory located in any UTHSC-H faciity work with human blood, tissue, cells microorganisms, or recombinant DNA will be used?

○ Yes ◉ No

If so, please list location:

28.3 Does the research protocol involve the use of RADIOACTIVE MATERIALS or RADIATION PRODUCING DEVICES (x-rays or lasers)?

○ Yes ◉ No

List nuclides or device to be used:

28.4 Does the research protocol involve the use of designated HAZARDOUS CHEMICALS?

○ Yes ◉ No

List chemical(s) requiring review:

29.0 HIPAA - Guide to completing the appropriate forms - Exemption

29.1 * Will you be accessing Protected Health Information (PHI - any identifiable health information) from a covered entity?

◉ Yes ○ No

If **NO, DO NOT ANSWER** any questions in the second section of this page. Press **SAVE and CONTINUE** to go the next section of the application.

29.2 If YES, please continue to answer the next set of questions.

Will you be receiving de-identified information?

○ Yes ◉ No

If YES, will all 18 identifiers be removed?

○ Yes ◉ No

Will any PHI in your possession be coded?

◉ Yes ○ No

Will you have the code in your possession?

(•) Yes ( ) No

30.0 HIPAA - Guide to completing the appropriate forms - Waiver for Decedent Data, Retrospective Chart Review or Preparatory to Research

30.1 * Is the use or disclosure being sought solely for research on the PHI of those who are deceased?

( ) Yes (•) No

At the request of the covered entity, can you provide **documentation of the death** of the individuals whose PHI is being sought?

( ) Yes (•) No

30.2 * Is this a retrospective chart review?

( ) Yes (•) No

30.3 * Is the information you are obtaining solely to prepare a research protocol?

( ) Yes (•) No

If YES, Will you be removing PHI from the site (covered entity)?

( ) Yes (•) No

31.0 Authorization for Disclosure of Protected Health Information (PHI)

31.1

Based on your answers to the "HIPAA - Guide to completing the appropriate forms" section of the application, your study requires that you complete the **"Authorization for Disclosure of Protected Health Information (PHI) for Research"** form when you submit your application to the CPHS.

When you are in the initial submission form of iRIS, you can select and complete this form from the "Review Board Forms" section of the form.

32.0 Statement of Investigator

32.1 * Are you a STUDENT at UT Health Scence Center and submitting this proposal as part of a degree program (e.g. Masters, Doctorate, or other)?

( ) Yes (•) No

32.2 * "I have discussed the protocol with all of my collaborators. The research is NOT underway and WILL NOT BEGIN until approved by the CPHS."

(•) Agree ( ) Disagree
