# Supplementary material for: Safety and Tolerability of Lactobacillus reuteri DSM 17938 and Effects on Biomarkers in Healthy Adults: Results from a Randomized Masked Trial
Source: PLoS One. 2012 Sep 6;7(9):e43910. doi: 10.1371/journal.pone.0043910 (PMC3435331; doi:10.1371/journal.pone.0043910)
Supplement: Materials and Methods S1 — Detailed materials and methods for measuring the expression of TLRs, percentage of Tregs in PBMC by flow cytometric analysis, cytokine production by stimulated PBMC, fecal calprotectin, and microbiota analysis. (DOCX) [file pone.0043910.s003.docx]

**S3. Materials and Methods**

*Antibodies.* The following human antibodies were used for immunostaining of peripheral blood mononuclear cells (B cells, monocytes, dendritic cells) and TLRs: Human CD4 FITC Conjugate (Invitrogen, Camarillo, CA), Human CD25 R-Phycoerythryin conjugate, (Invitrogen, Camarillo, CA), Human CD14 FITC Conjugate (Invitrogen, Camarillo, CA), Human CD19 FITC Conjugate (Invitrogen, Camarillo, CA), lin-1 (C3, 14, 16, 19,20, 56) FITC (BD Bioscience; San Jose, CA), human CD1cAPC (BDCA-1)-APC (Miltenyi Biotec, Auburn, CA), CD303 (BDCA-2)-APC (Miltenyi Biotec, Auburn, CA), PE anti-human CD282 (TLR2) ( BioLegend,SanDiego, CA), PE Conjugated Anti-human TLR-4 (CD284) (eBioscience, San Diego, CA).

The following anti-human mouse antibodies were used for isotype controls: PE Mouse IgG2a, K Isotype Control (eBioscience, San Diego, CA), PE Mouse IgG2a, K Isotype (BioLegend, San Diego, CA), APC Conjugated Mouse IgG2a, K Isotype Control (eBioscience, San Diego, CA). Alexa Fluor 647 Foxp3 (eBioscience, San Diego, CA) was used for staining of Tregs.

*Expression of TLR2 and TLR4 in human peripheral blood mononuclear cells (PBMC).* Whole blood samples were aliquoted into 300 µl suspensions in flow cytometric plastic assay tubes and stained with cell surface antibodies noted above. Isotype control staining was also performed. Samples were incubated at 4^o^ C for 30 minutes then washed with 1 ml of PBS-EDTA. Fixation-Permeablization Buffer (eBioscience, San Diego, CA) added to samples, which were then incubated and stored at 4^o^ C for 60 minutes. After incubation, the cell lysate was washed three times with 1 ml of phosphate-buffered saline (PBS). After the last wash, cells were resuspended in 300 µl of PBS and incubated at 4^o^ C in preparation for flow cytometric analysis.

*Percentage of Treg cells.* PBMC were isolated from whole blood by Ficoll-Paque (GE Healthcare, Cat#17-5442-02). PBMCs were treated with CD4 FITC and CD25 PE surface stains and incubated at 4^o^ C for 60 minutes. Following incubation, PBMCs were washed with 1 ml of PBS-EDTA. One ml fixation-permeablization buffer was added to PBMCs, and sample was incubated for another 60 minutes at 4^o^ C. Following incubation, samples were washed twice with 500 µl of permeabilization buffer (eBioscience, San Diego). After washing, cells were permeabilized with permeabilization buffer and stained with 5 μl (0.06 μg) of AF 647 Foxp3. Cells were then incubated at 4^o^ C for 60 minutes. After incubation, cells were again washed once with 500 µl of permeabilization buffer. Cells were resuspended in 300 µl of PBS and stored at 4^o^ C in preparation for flow cytometric analysis.

*Flow Cytometric Analysis and Cell Sorting.* Immunofluorescence staining was performed after washing the cells with PBS and resuspension in PBS. Cells were incubated at 4^o^ C until analyzed by flow cytometry (FACS Caliber; Becton Dickinson). Immunostained lymphocytes, subsets of T cells, B cells, monocytes, and dendritic cells were sorted with FlowJo software (TreeStar, Inc). Mean fluorescence intensity (MFI) for TLR was calculated by FlowJo software.

*Cytokine Production by Stimulated PBMC.* Peripheral blood mononuclear cells (PBMC) were isolated from blood samples taken at baseline, at 2 months of treatment, and at 4 months after completion of treatment using Ficoll-Paque^®^ PREMIUM (GE Healthcare, Pittsburgh, PA). Purified PBMC (2 x 10^6^ cells/mL) in serum-free XVIVO-15 medium (BioWhittaker, Gaithersburg, MD) were incubated with stimulants in a final volume of 0.2 mL per well in triplicate in 96-well plates (Corning^®^ Costar^®^, Lowell, MA ) in 5 % CO_2_ at 37^o^C. The agonists were phorbol 12-myristate 13-acetate (PMA) (Sigma-Aldrich^®^, St. Louis, MO) at 0.025 μg/mL plus ionomycin (Sigma-Aldrich^®^) at 1 μg/mL. Cell culture supernatants were collected after 16 hours of stimulation and stored at -80°C prior to analysis. Thawed supernatants were subsequently analyzed using the MSD^®^ Human ProInflammatory 9-Plex Tissue Culture Kit (Meso Scale Discovery®, Gaithersburg, MD); each well of the 96-well-plate contained antibodies to human IL-2, IL-8, IL-12p70, IL-1β, GM-CSF, IFN-γ, IL-6, IL-10, and TNF-α. Calibration curves were prepared with a range of 0-10,000 pg/mL. The assays were run according to the manufacturer’s instructions. Results from cytokines were expressed as pg/mL of cell culture supernatant.

*Fecal Calprotectin.* Stool samples were prepared and analyzed according to the manufacturer’s instructions (PhiCal^TM^ Test, Eurospital SpA, Trieste, Italy). Stool was collected in screw-capped plastic containers, the weight of each sample (80-120 mg) was measured, and an extraction buffer containing citrate and urea was added in a weight per volume ratio of 1:50. The samples were mixed for 30s by vortex and homogenized for 30 min. One ml was transferred to a tube and centrifuged for 20 minutes. The supernatant was collected and analyzed with PhiCal^TM^ Test, a quantitative calprotectin ELISA, for determination of calprotectin. The initial dilution of extract was 1:50. Further dilutions of high concentration samples were done if the readings were off-scale. Calprotectin results were calculated and expressed as µg/g of stool weight.

*DNA extraction.* DNA was isolated from 180 mg samples of stool specimens using a commercial kit following the manufacturer's instructions for isolation of DNA from gram positive bacteria (QIAamp DNA Mini Kit, Qiagen, Valencia, CA).

*Changes in stool microbiota.* Analysis of banding patterns on denaturing gradient gels (DGGE) was used to assess whether LR alters bacterial composition in the gut. Bands that migrated to a position in the gradient near that of the probiotic control were sequenced to confirm species identity. PCR-amplification of 16sRNA gene sequences from stool DNA, DNA sequencing and DGGE analyses were performed as previously described[^38^](#_ENREF_38)^,^ [^39^](#_ENREF_39). The number of patients whose DGGE banding pattern changed was determined for each sampling interval in both the LR and placebo-treated control group.

*Quantification of LR.* A TaqMan quantitative polymerase chain reaction (qPCR) assay targeting the *L. reuteri* 16S rRNA gene was used to quantify LR in stool DNA. Details of the assay, along with primer and probe sequences, have been described in detail elsewhere[^39^](#_ENREF_39). Briefly, triplicate standard curves were prepared using 10-fold serially diluted LR genomic DNA over a concentration range of 0 to 10^7^ genomes per qPCR. Five ng of template stool DNA was used in each qPCR and threshold cycle (Ct) values were used to calculate LR concentrations and assess the relative abundance of LR in stool specimens. A CFX96-Real Time PCR Detection System (Bio-Rad Laboratories, Hercules, CA) was used to perform qPCR assays. The qPCR conditions were, 2 min at 50^o^C and 10 min at 95^o^C, followed by 45 cycles of 15s at 95^o^C and 1 min at 60^o^C. The stool DNA samples used for qPCR analysis were the same as those described for DGGE analysis above.
